# How Japan Took the Lead in the Race to Discover Element 119

**DOI:** 10.1021/acscentsci.4c01266

**Published:** 2024-08-15

**Authors:** Felicity Nelson

At the start of the new year, nuclear chemists Hiromitsu Haba and Kouji Morimoto slide precisely 119 Japanese yen into the collection box at their local shrine. They are seeking good fortune in their hunt for an elusive entity: element 119.

Haba and Morimoto are part of a research team at Riken Nishina Center for Accelerator-Based Science, just outside of Tokyo. The team has spent the past 4 years using a particle accelerator to smash atoms together at high speeds in a bid to synthesize the new element.

Once created, element 119 will contain 119 protons—the most of any element discovered. It will sit on a new, eighth row of the periodic table, and it could be the first element to be named since 2016.

The US, Russia, China and Germany also developing powerful experimental setups to search for elements 119 and 120. But because of a mix of geopolitics, strategy, and luck, Japan has unexpectedly takenthe lead in this race to discover a new element.

## “An impossible dream”

Japan has tasted defeat in the element-hunting game once before: In 1908, the discovery of its namesake element, nipponium, was announced by Japanese chemist Masataka Ogawa. But nipponium was never added to the periodic table of elements because Ogawa had placed it in the position of element 43 instead of element 75, and, by the time the mistake was realized, element 75 had already been discovered by German chemists and named rhenium. So it was the culmination of more than a century of element hunting when Riken scientists announced the discovery of element 113 in 2012.

Riken’s lead physicist on the project, Kosuke Morita, needed all the luck he could get during his 9-year search for element 113. He was careful to observe certain superstitions: traveling on national route 113, riding the No. 113 Shinkansen Nozomi bullet train, and donating 113 Japanese yen at every shrine he visited. “I used to do it very seriously,” he says. “We did everything we could do as researchers. All that remained was to pray to God.”

After around 4 trillion atomic collisions, just three atoms of element 113 were detected, and each existed for around 2 ms before breaking apart. But that was enough. Element 113 was the first element to have been discovered in Asia. It was given the name nihonium after Nihon—a name for Japan that translates to “land of the rising sun.”

Hideto En’yo, Riken’s then director, wrote that the discovery of a new element had been “an impossible dream for Japanese people” and that “thereafter, Japanese became fanatics of the Periodic Table” (*Pure Appl. Chem.* 2019, DOI: 10.1515/pac-2019-0810).

Nihonium became a conversation starter for Japanese ambassadors. Element research made it into Japanese comic books. Wako̅, Japan, the city where Riken is based, decorated Nihonium Avenue with 118 bronze pavestones, one for each of the known elements of the periodic table. “Wako City is now officially the city of elements,” En’yo wrote. Buoyed by their success with nihonium, the Japanese researchers at Riken have now turned their attention to element 119.

But Riken’s success with nihonium wasn’t a shoo-in. The center was racing toward the confirmation of element 113 against the GSI Helmholtz Centre for Heavy Ion Research in Germany and a three-way collaboration between Lawrence Livermore National Laboratory (LLNL) and Oak Ridge National Laboratory (ORNL) in the US and the Joint Institute for Nuclear Research (JINR) in Russia—institutions that are now joined by the US’s Lawrence Berkeley National Laboratory (LBNL) as the major players attempting to synthesize elements 119 and 120.

**Figure:**
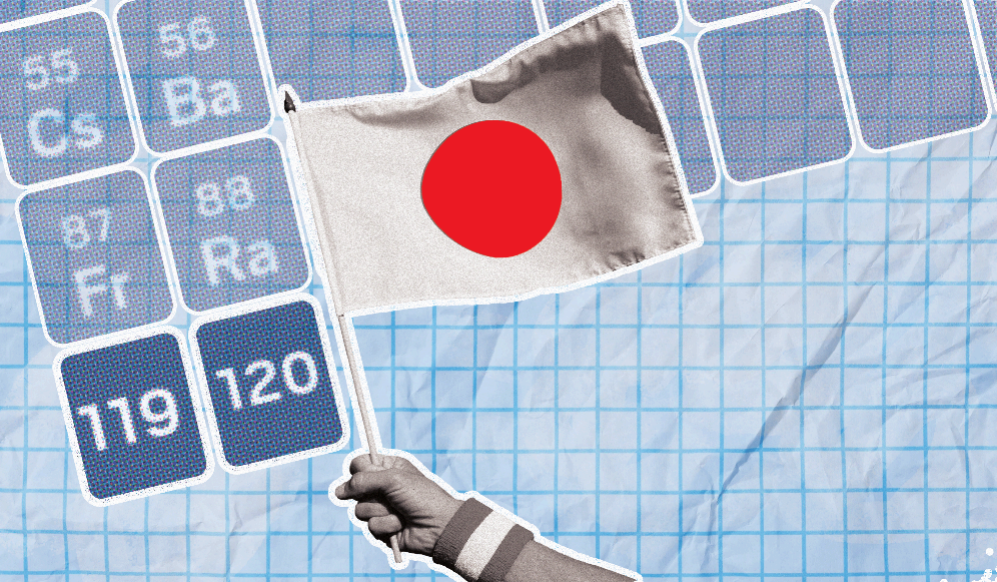
Credit: Madeline Monroe/C&EN/Alamy.

## Taking the lead

In 2017, Riken started to upgrade its facilities as it embarked on its search for element 119; this happened just as one of the research team’s biggest competitors, the prolific LLNL-ORNL-JINR collaboration, broke down. That year, LLNL’s request to renew a longstanding memorandum of understanding with the Russian institution was denied by the US Department of State.

“We had to send this really horrible email to [the scientists in Russia] that basically said, We’re sorry, but after all this time, we’re no longer allowed to work with you,” says nuclear chemist Dawn Shaughnessy who led this research at LLNL. “It was very heartbreaking because we’re scientists and we just want to do science.”

JINR’s Flerov Laboratory of Nuclear Reactions invested $60 million into a new facility in Dubna, Russia, called the Superheavy Element Factory, which was due to begin experiments to create element 119 in 2019. But political decisions made by the Russian government—including the enactment of increasingly repressive laws against organizations labeled “foreign agents” since 2012 and the invasion of Ukraine in February 2022—made it difficult for Russian scientists to collaborate internationally, import materials and equipment, source funding, attend conferences, or coauthorpapers with scientists based overseas.

“The situation is actually not easy in the current turbulent environment,” JINR lead scientist Yuri Oganessian told *Chemistry World* last year. Following US sanctions, JINR “cannot cooperate with Oak Ridge and Livermore because JINR is located in Russia,” he said. *C&EN* received no response from JINR by email, and the JINR press office hung up on both of *C&EN*’s calls.

## The US goes after element 120

After a 50-year drought in element discoveries at LBNL, the institution is preparing to use one of its cyclotrons to discover element 120, pitting the US against China, Russia, and Germany.

LBNL was “working already towards [creating element 120], independent of the geopolitical situation,” says Reiner Kruecken, director of the Nuclear Science Division at LBNL. The decision to pursue element 120 had been made a few years ago, after theoretical predictions indicated that there was a sufficiently high probability of collisions between nuclei, he says.

Despite being a few dozen kilometers apart, LBNL and LLNL hadn’t partnered on an element discovery for decades—LBNL had vied with Russia’s JINR throughout the 20th century, while LLNL had partnered with JINR starting in the late 1980s. But today, the two US laboratories are official collaborators on the element 120 project, and the creation of new elements now features in the US’s agenda for nuclear science: a 2022 white paper by the US Heavy Element program identified LBNL’s cyclotron as “presently best suited to attempt a new element search.”

**Figure:**
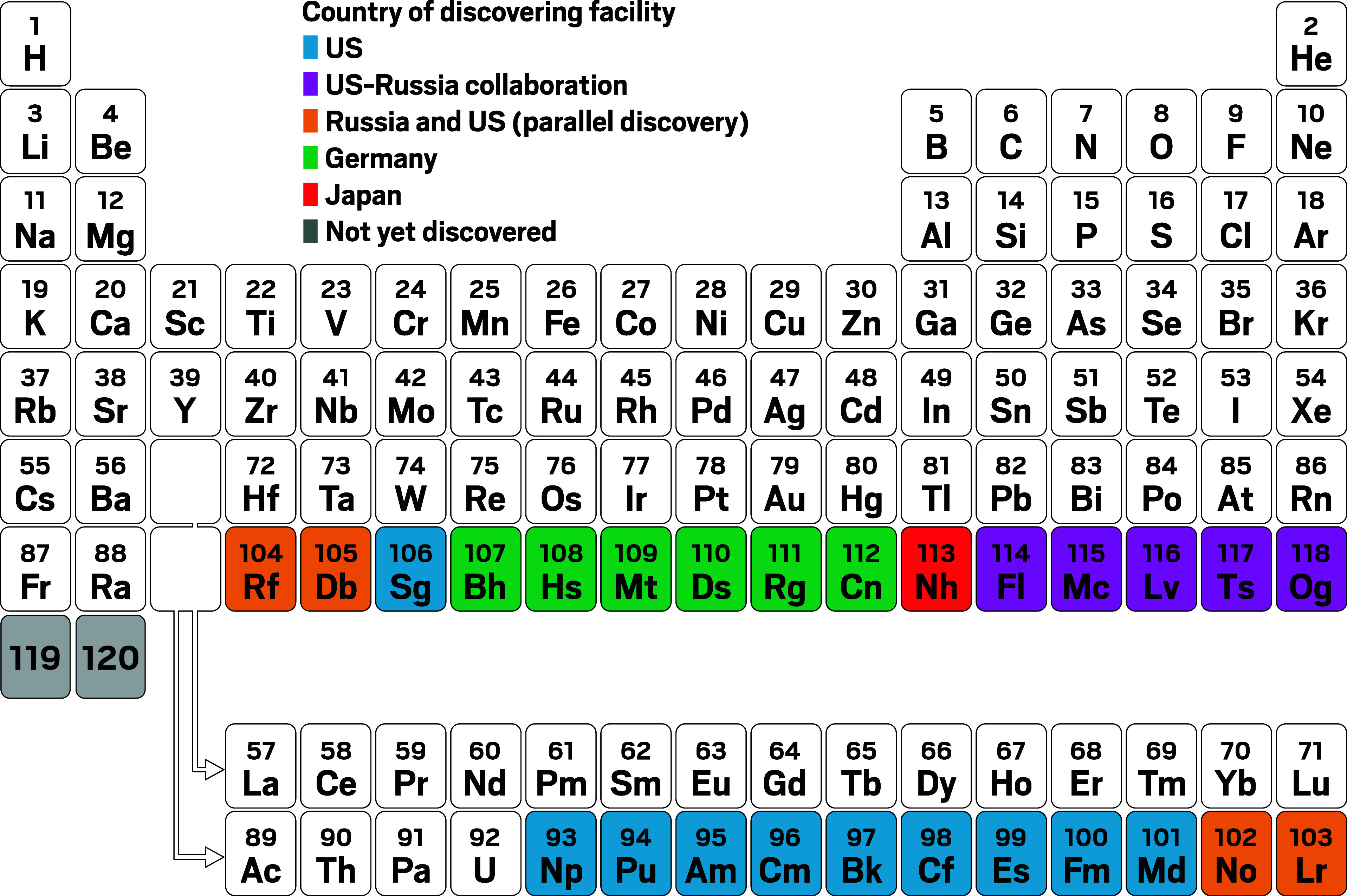
Of the past 26 elements created, Japan’s Riken has discovered only one. Credit: Adapted from *Science*.

## Next-generation equipment

Creating a superheavy element through nuclear fusion is like winning the lottery, says nuclear chemist Christoph Düllmann, who leads the superheavy element chemistry research at the GSI.

The heavier the atoms get, the more positively charged protons they have and the more energy—and attempts—it takes to overcome those protons’ repulsion and get nuclei close enough together to fuse. Beyond element 118, the probability of two heavy nuclei fusing to form a new element is so low, “you either need a lot of time or a lot of particles,” says Shaughnessy.

To buy more proverbial lottery tickets, teams around the world are renovating their hardware. The research teams in Japan, Russia, China, Germany, and the US have been boosting the intensity of their ion beams and can now fire more than 6 trillion atoms per second at a target, an upgrade that is akin to replacing a kitchen tap with a fire hose.

In Japan, at Riken, a superconducting electron-cyclotron-resonance ion source (SC-ECRIS) and the superconducting Riken linear accelerator (SRILAC) were constructed to create a beam with five times the current of that used in the nihonium experiment. A labyrinth of machines turns metal into plasma using a high-temperature oven and then accelerates many charged particles at once to roughly one-tenth the speed of light.

This intense beam is fired at samples of target material that have been embedded into a metal wheel. This wheel rotates about 2,000 times per minute, which prevents the beam from incinerating it. Even at these high speeds, the target reaches temperatures of 500–1,000 °C.

About once every 200 days, two nuclei will collide and fuse together. The resulting superheavy atom pops out the back of the target, which is as thin as foil.

Another constraint is time. As elements get heavier, they generally become less stable and have shorter half-lives. The discovery of element 119 therefore depends on efficient components to separate the ions streaming out of the accelerator from the superheavy atoms. “Everything just has to be a little bit better to get to these next set of elements,” says Shaughnessy. Toward that goal, Riken researchers have built a new gas-filled recoil ion separator called GARIS-II that uses five magnets to collect the products of fusion reactions with twice the efficiency of the previous model.

After they are separated from the ion beam, superheavy atoms are sent to silicon strip detectors, where they throw out α particles and decay into lighter elements within milliseconds. The Riken detectors can record the charge signals from these reactions more than once per nanosecond, and scientists can work backward from these data to identify whether element 119 was the parent atom.

In 2018, Riken started using its existing ring cyclotron and linear accelerator (RILAC) to run exploratory experiments while SC-ECRIS and SRILAC were being constructed. The new setup was completed in 2020, but the COVID-19 pandemic and a breakdown of the 40-year-old RILAC caused work interruptions. The researchers in Japan are now running the element 119 experiment 24/7.

**Figure:**
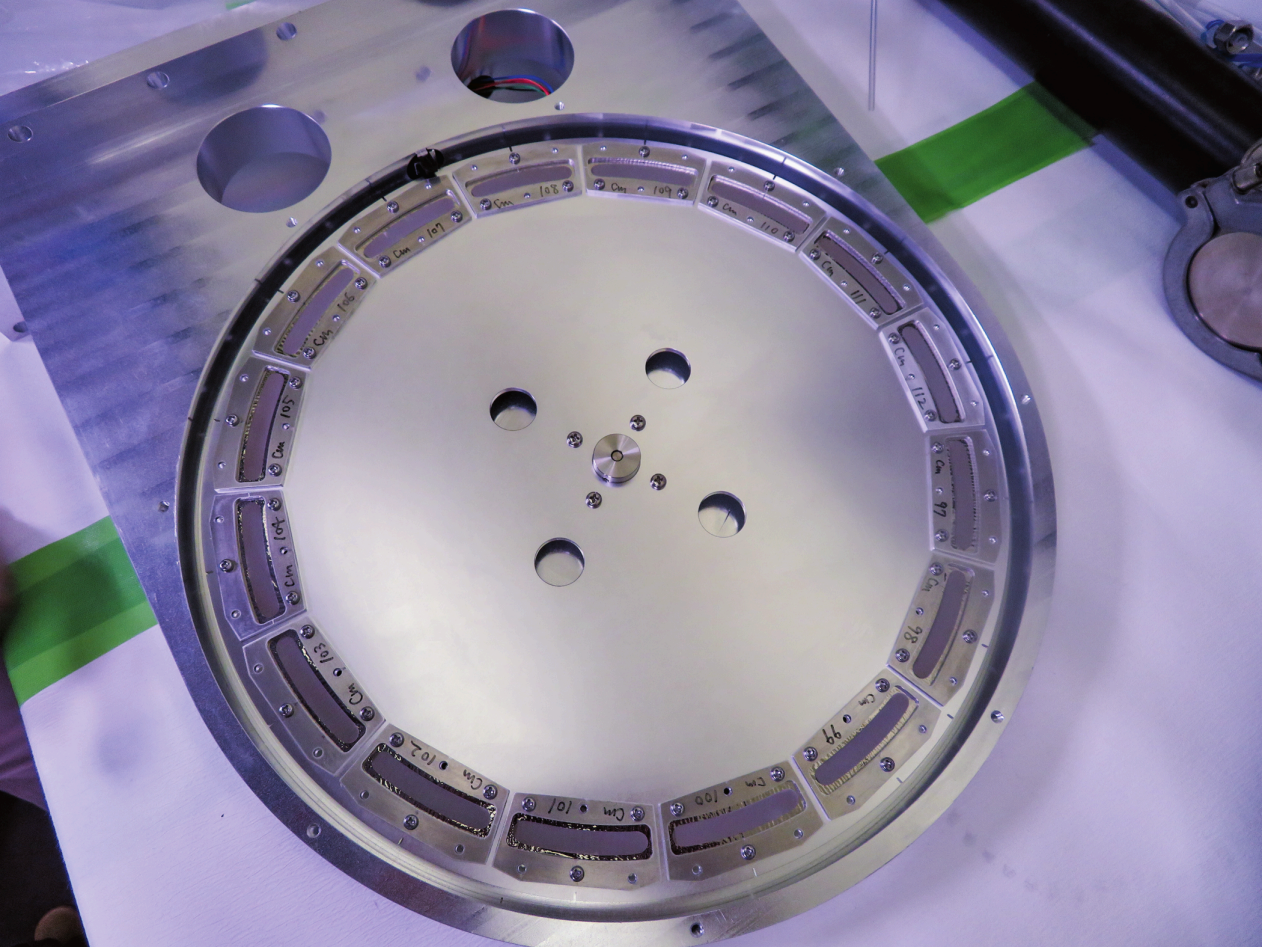
Riken’s aluminum target wheel has 16 curium oxide target samples placed around the rim. Credit: Riken.

## Picking a winner

Some of Riken’s choices of experimental setup are constrained by the realities of element hunting. For example, they are committed to using a hot fusion reaction—a process with relatively high excitation energy—to make element 119 because a lower-energy cold fusion reaction like the one that created nihonium would take too long.

And the workhorse of hot fusion, a calcium-48 ion beam, won’t work either. The isotope boasts a high number of neutrons and highly stable numbers of both neutrons and protons in its nucleus, creating a doubly magic isotope. Together, these properties make it especially good at hot fusion reactions. Unfortunately for the Riken team, calcium-48’s fusion partner would be einsteinium, a metal currently produced by bombarding curium rods with neutrons. This is a very slow process, so it’s time intensive to gather even a few micrograms.

**Figure:**
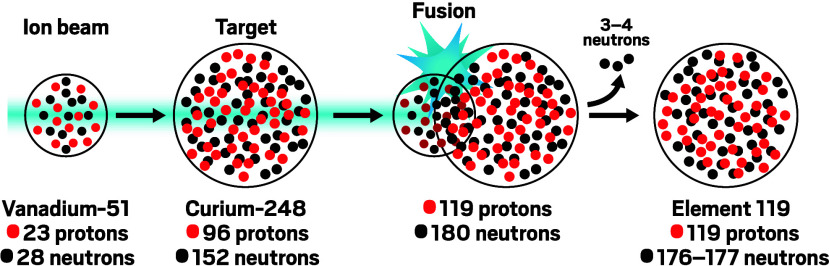
To make element 119, RIKEN researchers are smashing vanadium ions into curium nuclei. The so-called hot fusion reaction releases a relatively high number of neutrons.

With einsteinium plus calcium off the table, three other combinations of nuclei are being considered as hot-fusion candidates to create element 119. Each pairing produces an atom with 119 protons:

- Americium-243 plus a chromium-54 beam
- Curium-248 plus a vanadium-51 beam
- Berkelium-249 plus a titanium-50 beam

So far, Riken and its collaborators have used only one combination of nuclei—curium-248 and vanadium-51—while the JINR and the Institute of Modern Physics of the Chinese Academy of Sciences in Lanzhou, China, are each planning to use multiple combinations of beams and targets.

Riken chose the curium–vanadium pair because both elements are easy to manipulate in terms of their radiation safety and chemical properties. Plus, curium-248 is easier to prepare than americium-243 or berkelium-249, and vanadium-51 is cheaper and more available than titanium-50.

Another major downside to using berkelium-249 is that its 327-day half-life is much shorter than the half-lives of curium-248 and americium-243. “After a year, you have less than half the material,” says physicist Krzysztof Rykaczewski, a senior researcher at ORNL, which supplied the curium-248 for the Riken experiment. But the upside of berkelium-249 plus titanium-50 is that the probability of two atoms colliding and interacting is slightly higher than with curium-248 plus vanadium-51, a consequence of the nuclei in the former pair having a smaller force of repulsion.

GSI ran a 4-month experiment using berkelium-249 plus titanium-50 in 2012, but they did not find any atoms of element 119. The facility could not continue this research: its resources were put toward constructing the €3.3 billion (about $3.6 billion) Facility for Antiproton and Ion Research. GSI researchers are now focused on smaller-scale experiments that can answer fundamental questions such as, What are the best experimental conditions for finding element 120?

LBNL might be ahead of the game there, though. In July, LBNL researchers made history by becoming the first team to create element 116 using a titanium-50 beam—a proof-of-concept study that is an “essential precursor” to creating element 120 using a titanium-50 beam, according to a preprint article on the arXiv server. Based on this result, LBNL predicts that element 120 could be created in around 220 days. LBNL is planning to use a californium-249 target, which can be produced in high-enough quantities by ORNL, and a beam of titanium-50, which is reasonably stable thanks to its magic number of neutrons.

## The island of stability

Any atom of element 119 or 120 will likely last only a few milliseconds before emitting bundles of protons and neutrons and decaying to lighter elements.

Such a fragile and fleeting entity is unlikely to be practically useful. But by pushing the limits of the periodic table, researchers hope to better understand the nature of atoms and nuclei—structures that make up all visible matter. Researchers also predict that some isotopes of element 119 will reach the fabled island of stability and have long-enough half-lives to be used in applications.

When technetium, the first human-made element, was created in 1937, “no one knew what use it would be,” says Haba, the nuclear chemist leading the search for element 119 at Riken. Today, technetium-99m, which has a half-life of 6h, is used to detect the spread of bone cancer and to assess cerebral blood flow and the function of the thyroid, heart, and liver.

“Element 119 may also be useful in our daily life in 100 years,” says Haba.

## Felicity Nelson is a freelance contributor to

Chemical & Engineering News, *the independent news outlet of the American Chemical Society*.

